# Insights into Facilitated Subcutaneous Immunoglobulin Use in Patients with Secondary Immunodeficiency Diseases: A FIGARO Subgroup Analysis

**DOI:** 10.3390/cancers15184524

**Published:** 2023-09-12

**Authors:** Maria Dimou, Matthaios Speletas, Cinzia Milito, Aleksandra Pyzik, Dörte Huscher, Marta Kamieniak, David Pittrow, Michael Borte

**Affiliations:** 1 First Department of Propaedeutic Internal Medicine, National & Kapodistrian University of Athens Medical School, General Hospital “LAIKO”, 11527 Athens, Greece; 2 School of Health Sciences, Department of Immunology and Histocompatibility, Faculty of Medicine, University of Thessaly, GR-41500 Larissa, Greece; speletas@gmail.com; 3 Department of Molecular Medicine, Sapienza University of Rome, 00161 Rome, Italy; cinzia.milito@uniroma1.it; 4 Department of Clinical Immunology, Center of Oncology St. Jana, 20-090 Lublin, Poland; aleksandra.pyzik@gmail.com; 5 Institute of Biometry and Clinical Epidemiology, Berlin Institute of Health, Charité-Universitätsmedizin Berlin, 10117 Berlin, Germany; dhuscher@posteo.de; 6 Takeda Development Center Americas, Inc., Cambridge, MA 02142, USA; marta.kamieniak@takeda.com; 7 Institute for Clinical Pharmacology, Medical Faculty, Technical University of Dresden, 01307 Dresden, Germany; david.pittrow@tu-dresden.de; 8 Innovation Center Real World Evidence, GWT-TUD GmbH, 01067 Dresden, Germany; 9 Hospital for Children and Adolescents, St. Georg Hospital, Academic Teaching Hospital of the University of Leipzig, IDCL (ImmunoDeficiency Center Leipzig), 04129 Leipzig, Germany; michael.borte@sanktgeorg.de

**Keywords:** facilitated subcutaneous immunoglobulin, immunoglobulin replacement therapy, secondary immunodeficiency diseases, real-world study, self-administration

## Abstract

**Simple Summary:**

The prospective, observation Facilitated Immunoglobulin Administration Registry And Outcomes (FIGARO) study was conducted to assess the clinical use and tolerability of facilitated subcutaneous immunoglobulin (fSCIG). In this report, final data for the cohort of patients with secondary immunodeficiency (SID) (*n* = 31, mean age 61.4 years) are presented. The median monthly dose of fSCIG (30 g) and median monthly infusion volume per patient (300 mL) were constant and serum immunoglobulin (Ig) trough levels increased over the observation period (36 months). Most patients required one infusion site and received the full dose every 3–4 weeks. Infusion site inflammation or headache were reported at inclusion (*n* = 1 each). No adverse drug reactions were reported during follow-up. The observed efficacy and tolerability of fSCIG and the possibility of self-administration at home demonstrated that fSCIG positively influenced the treatment experience for patients with SID, most of whom were elderly with a high comorbidity burden.

**Abstract:**

The Facilitated Immunoglobulin Administration Registry And Outcomes (FIGARO) Study was a European, multicenter, prospective, observational study conducted across Europe designed to provide insights on the clinical use and tolerability of facilitated subcutaneous immunoglobulin (fSCIG). Data herein are reported for the cohort of patients with secondary immunodeficiency (SID), with a subgroup analysis by age. The SID cohort included 31 patients: 1 pediatric, 15 adult, and 15 older adult patients. Over the 36-month observation period, the median monthly dose of fSCIG (30 g) and median monthly infusion volume per patient (300 mL) remained constant in both adult-age cohorts. Serum trough levels tended to increase over time. Most patients required only one infusion site and could receive the full dose every 3–4 weeks. There was a trend toward self-administration at home. In the adult group, infusion site inflammation and headache were reported at the inclusion visit (*n* = 1 each), with no adverse drug reactions reported at any of the follow-up visits. No acute severe bacterial infections were reported during the study follow-up. These results demonstrate the feasibility and tolerability of fSCIG use in patients with SID and the flexibility of administration settings including self-administration at home in patients aged ≥65 years.

## 1. Introduction

Secondary immunodeficiencies (SIDs) are acquired immunodeficiencies caused by external factors including underlying hematological diseases and their treatments, transplantation, and use of certain medications or immunosuppressive agents [1,2,3,4]. B-cell malignancies, including chronic lymphocytic leukemia (CLL) and multiple myeloma (MM), are associated with SID, and mainly present with hypogammaglobulinemia, characterized by low serum immunoglobulin (Ig) levels [5]. Infections are a common occurrence among patients with SID [6], and recurrent infections can lead to end organ damage and death [2,7]. A full immunological workup, involving clinical, radiological, and laboratory information, is required to assess potential susceptibility to infections [4,8]. Delays in diagnosis and reactive treatment approaches diminish patient health-related quality of life (HRQoL) and increase mortality [5].

Ig replacement therapy (IGRT) can reduce the risk of infections in immunodeficiency disorders [8,9]. Intravenous (IV) and subcutaneous (SC) routes of administration of IGRT (IVIG and SCIG, respectively) are both used and have comparable efficacy [10,11], but fewer adverse drug reactions (ADRs) have been associated with SCIG and venous access is not required for this method [10]. Administration of SCIG is less time consuming than IVIG and allows for more flexible and convenient home-based self-administration, which has been reported to improve various aspects of patient HRQoL including general health, pain, and vitality [12]. Pharmacokinetics of SCIG may be more favorable, as treatment leads to higher and more stable immunoglobulin G (IgG) trough levels, providing patients with more consistent protection against infections [13]. However, an important caveat of conventional SCIG (cSCIG) is that a limited volume can be infused into SC tissue, necessitating weekly or biweekly administration across multiple (2–4) infusion sites [10,14]. Consensus and recommendations on the use of IGRT in SID are sparse [15,16].

Hyaluronidase is an enzyme that has been implemented into various SC medicines to help increase the volume that can be delivered through this mode of administration by improving dispersion and absorption [17]. Facilitated SCIG (fSCIG) applies this approach by combining recombinant human hyaluronidase (rHuPH20) and 10% human normal IgG in a dual-vial unit [18]. rHuPH20 acts to depolymerize hyaluronan, which allows infusion of larger volumes of IgG than is possible with cSCIG [10,14,18,19,20]. fSCIG combines the benefits of IVIG and cSCIG, and is approved in the European Union as replacement therapy in primary immunodeficiency disease (PID) patients of all age groups, and in patients with secondary immunodeficiency diseases (SIDs) who suffer from severe or recurrent infections, ineffective antimicrobial treatment, and either proven specific antibody failure or serum IgG level of <4 g/L [18]. The safety and efficacy of fSCIG in patients with PID are well established [14,18,21,22,23,24]; clinical data on the use of fSCIG in patients with SID are limited [25].

The Facilitated Immunoglobulin Administration Registry And Outcomes (FIGARO) study was a multicenter, prospective, observational study conducted with the support and oversight of the European Society for Immunodeficiencies (ESID). It was designed to provide insights on the real-world utilization and tolerability of fSCIG in patients with PID and SID. Reported here are the final data for the cohort of patients with SID, together with subgroup analysis by age to assess the impact of older age on fSCIG use.

## 2. Materials and Methods

### 2.1. Study Design

FIGARO was a phase 4 study conducted in 14 centers in 6 European countries: Czech Republic, Germany, Greece, Italy, Poland, and Spain. Study initiation was December 2016, and database closure was August 2021. Physicians in hospitals or office-based settings were eligible to participate if they had experience in treating patients with IgG and were responsible for treating patients with fSCIG. Data collection was prospective (retrospective documentation of anamnestic information) using information based on patient charts, diaries, or patient interviews. Patients were followed for up to 36 months, and follow-up visits were allocated into 6-month intervals over the observation period. In some cases, multiple visits were combined for 1 interval. Full details of the FIGARO study design have been published [26].

### 2.2. Patients

Eligibility criteria for the FIGARO study have been previously described [21]. Patients of all age groups who had received or were prescribed at least 1 dose of fSCIG infusion for PID or SID had an indication for chronic IgG treatment, were available for long-term documentation, and provided informed consent were eligible for inclusion in the FIGARO study. Non-eligibility criteria were not defined to minimize selection bias. This analysis included patients in the SID cohort of FIGARO.

### 2.3. Study Endpoints

Drug utilization pattern was assessed as the primary endpoint. Secondary endpoints included concomitant medications (defined according to ATC (Anatomical Therapeutic Chemical) code) and disease states (defined according to MedDRA (Medical Dictionary for Regulatory Activities) code), mean serum trough levels after fSCIG administration, rates of infections, training sessions, and nurse visits at home. Premedication prior to fSCIG infusion and ADRs were also assessed.

### 2.4. Statistical Analyses

The study size was determined by feasibility aspects. No formal sample size calculation was performed. Data were summarized using descriptive statistics and are presented as observed, with no imputations for missing values. Continuous variables were expressed as number of values, with averages presented as mean and standard deviation (SD) or median and interquartile range (IQR). Categorical variables were described as frequency counts (absolute and relative). Data were stratified by age into pediatric (<18 years), adult (18–64 years), and older adult (≥65 years) subgroups.

## 3. Results

### 3.1. Patients’ Demographics and Clinical Characteristics at Inclusion

**Figure 1:**
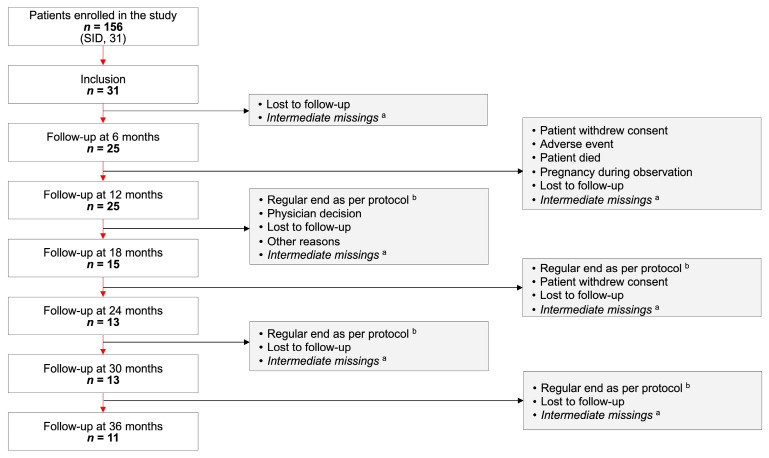
Patient disposition. ^a^ Visits between available visits are missing. ^b^ Patients were followed to May 2021 regardless of date of inclusion. SID, secondary immunodeficiency disease.

Of the 156 patients in the FIGARO study population, 31 patients had SID and were included in this analysis (Figure 1). The SID cohort included 1 pediatric patient, 15 adult patients, and 15 older adult patients. Patients attended 149 visits, ranging from 1 visit (inclusion only) to 12 follow-up visits per patient. Over the observation period, one patient withdrew consent, one patient died, and one patient discontinued due to physician decision to change treatment. Many patients in the SID cohort were recruited during the later stages of the study, such that at study close, depending on the date of inclusion, 12-, 24-, and 36-month follow-up data were available for 25, 13, and 11 patients, respectively (Figure 1).

At inclusion, the mean age of the patients was 61.4 years (range 3–88 years), 61.3% were male, and 93.5% were identified as Caucasian/White (Table 1). The most common indications for IgG treatment in adult patients were CLL (8/15, 53.3%) and indolent lymphoma (1/15, 6.7%), 6 of 15 (40.0%) (Table 1). All older adult patients received IgG treatment for CLL (12/15, 80%) or indolent lymphoma (3/15, 20%) (Table 1).

At inclusion, more than 80% of patients reported concomitant diseases, including arterial hypertension (41.9%), cancer (22.6%), gastrointestinal disease (19.4%), chronic obstructive pulmonary disease (12.9%), and hyperuricemia (12.9%).

At inclusion, almost all patients (93.5%) were receiving chemotherapy, immunosuppressive therapy, or supportive therapy. The most common chemotherapies included venetoclax (*n* = 10, 32.3%), ibrutinib (*n* = 4, 12.9%), and rituximab (*n* = 3, 9.7%). The most common supportive therapies included *Pneumocystis jirovecii* pneumonia (PJP) prophylaxis, virostatics, and antibiotics (Table 1). Older adult patients received PJP prophylaxis (66.7% vs. 26.7%) and virostatics (53.3% vs. 33.3%) more frequently than adult patients (Table 1).

Before study enrollment, all patients had received IVIG and/or SCIG (Table 1). The most frequently reported reasons for prior IGRT discontinuation were patient request (6/23 [26.1%] discontinuations) and tolerability issues (5/23 [21.7%] discontinuations). Tolerability problems resulted in 5/13 (38.5%) discontinuations in adult patients but in none of the nine discontinuations in older adult patients (Table 1).

### 3.2. fSCIG Dose and Administration

**Figure 2:**
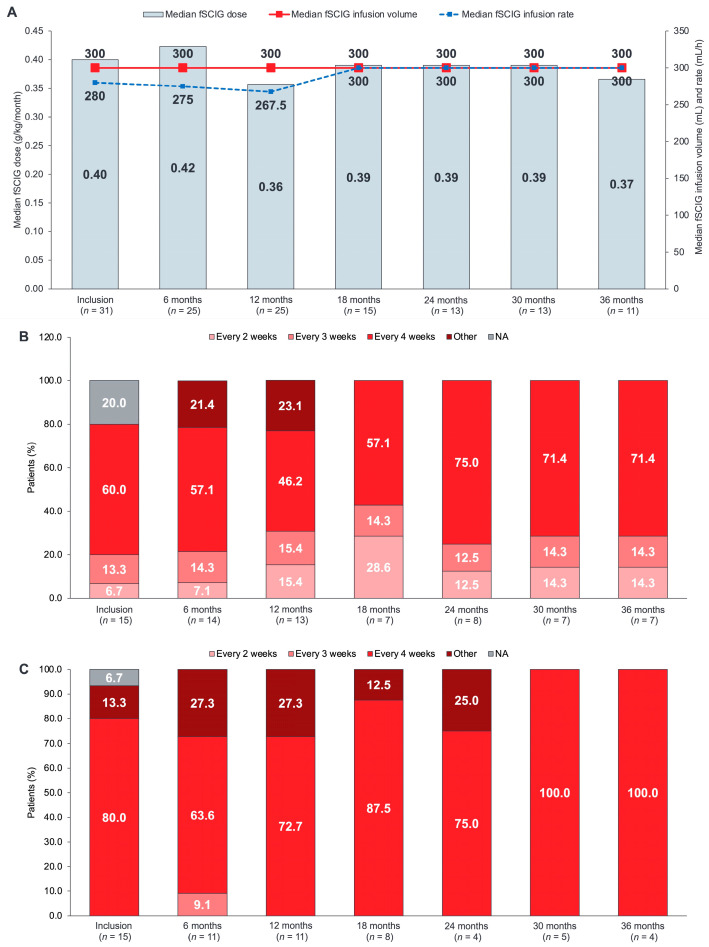
fSCIG infusion parameters and interval over 36 months of follow-up. (**A**) fSCIG dose, infusion volume, and infusion rate; (**B**) fSCIG infusion interval in patients 18–64 years of age; (**C**) fSCIG infusion interval in patients ≥65 years of age. *n* values represent number of patients at each visit; available values for each parameter may differ slightly due to missing data for that individual parameter. Other: includes treatment intervals of 7 weeks or longer. NA, not applicable as the patients had received only 1 fSCIG infusion to date. fSCIG, facilitated subcutaneous immunoglobulin; SID, secondary immunodeficiency disease.

The median monthly fSCIG dose during the most recent application at inclusion was 30 g (IQR 25–30 g) in the overall SID population, and in adult and older adult patients (Table 2). Over the 36-month observation period, the median monthly dose of fSCIG remained constant at 30 g (Figure 2). The median fSCIG dose by body weight at inclusion was 0.400 g/kg per month (IQR 0.333–0.500) and remained relatively constant over the course of the study (Table 2, Figure 2A). All patients received the full planned fSCIG dose at inclusion.

**Figure 3:**
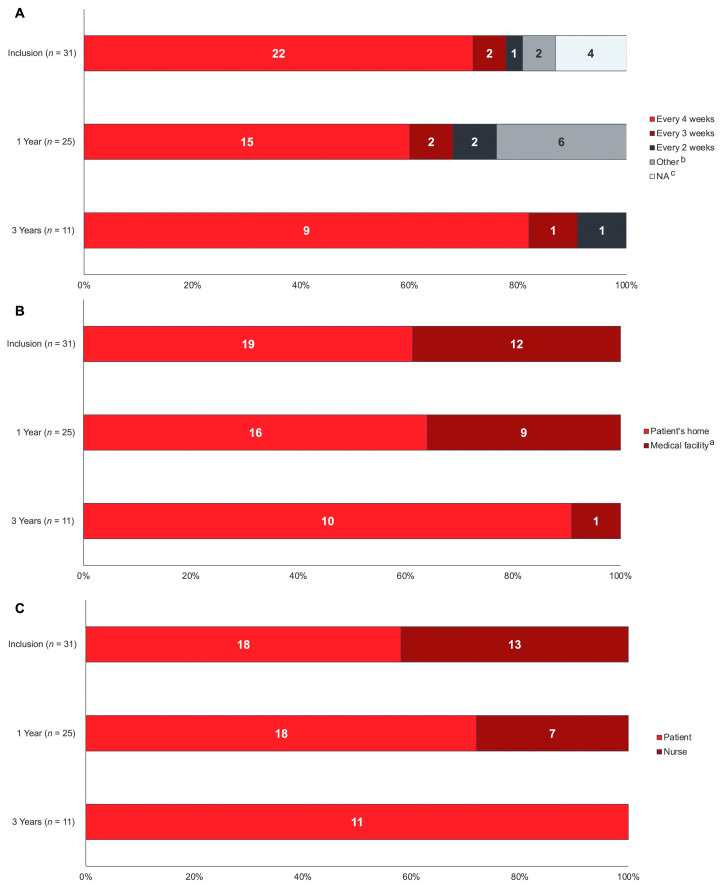
fSCIG administration at inclusion, 12 months, and 36 months. (**A**) Average treatment interval (**B**) location of fSCIG administration; (**C**) administrator of fSCIG infusion. ^a^ At inclusion, 1 year, and 3 years, respectively: doctor’s office (*n* = 1, *n* = 0, *n* = 0); hospital (*n* = 11, *n* = 9, *n* = 1). ^b^ Other: includes treatment intervals of 7 weeks or longer. ^c^ NA, not applicable as the patients had received only 1 fSCIG infusion to date. fSCIG, facilitated subcutaneous immunoglobulin.

Patients predominantly infused fSCIG every 3–4 weeks (24/31 (77.4%) patients at inclusion), with more patients moving to a 4-week infusion interval over the course of the study (9/11 (81.8%) patients at 36 months) (Figure 3A). Most patients (26/27 (96.3%)) infused into a single injection site (most commonly, the upper abdomen in adult patients (7/15, 46.7%) and lower abdomen in older adult patients (9/15, 60.0%)) (Table 2). At 36 months, 5/7 (71.4%) adult patients and 4/4 (100%) older adult patients achieved a 4-week treatment interval (Figure 2B,C).

At inclusion, 19 (61.3%) patients received their fSCIG infusion at home, and 18 (58.1%) patients self-administered (Figure 3B,C). At 12 and 36 months, more patients received their fSCIG infusion at home (16/25 (64.0%) and 10/11 (90.9%), respectively) and self-administered (18/25 (72.0%) and 11/11 (100.0%), respectively). Premedication with corticosteroids prior to fSCIG infusion was required by one patient at inclusion and another patient over the course of the study. Adherence to fSCIG treatment schedule was high, with all patients having available data infusing either as scheduled (28/30 (93.3%) patients) or within ±1–3 days (2/30 (6.7%) patients) of the scheduled date at inclusion. Throughout follow-up, almost all patients infused within 3 days of their scheduled dates.

At inclusion, serum IgG trough levels were reported in 15/30 (50.0%) patients. Median IgG serum trough level increased from 5.4 g/L (IQR 4.6–7.3 g/L) at inclusion to 6.5 g/L (IQR 4.1–7.3 g/L) at 12 months and 9.4 g/L (7.5–9.5 g/L) at 36 months.

### 3.3. fSCIG Infusion Parameters

The median per-patient fSCIG infusion volume was 300 mL (IQR 250–300 mL) at the time of inclusion and remained constant over the course of the study (Figure 2A). The median maximal infusion rate was 280 mL/h (IQR 240–300 mL/h) at inclusion, which increased to 300 mL/h (IQR 300–300 mL/h) at 18 months and stayed constant throughout the study. All patients used a 24.0-gauge needle, and all except one used a needle that was 12.0 mm in length. A single infusion site was used by all but one patient, and the site was most frequently in the upper or lower abdomen (Table 2). Almost all fSCIG infusions were administered using a pump (92.3–100% over the course of the study). Technical problems were rare (2/120 (1.7%) infusions). At the 6-month and 36-month follow-up assessments, one patient experienced a problem handling the pump. The full fSCIG dose was administered as planned for all but one infusion (131/132 infusions, 99.2%).

### 3.4. Safety

At inclusion, ADRs associated with the fSCIG infusion were reported for 2/31 patients (6.5%) in the overall SID population. One patient experienced infusion site inflammation and one patient reported a headache. No ADRs were reported at any of the follow-up visits. In total, 10/31 (32.3%) patients reported ≥1 acute severe bacterial infection (ASBI) events in the 12 months prior to inclusion, with no ASBI events reported throughout the study (Table S1). Pneumonia was the most common ASBI event (5/14 (35.7%) events) reported in the 12 months prior to inclusion, and it was only reported in older adult patients (5/15 (33.3%) patients).

Other bacterial infections in the 12 months prior to inclusion were reported in 11/31 (35.5%) patients in the overall SID cohort (Table S1). There were more other bacterial infections reported in the 12 months prior to inclusion in adult patients (19/27 (70.4%) events) than in older adult patients (8/27 (29.6%) events). During follow-up in the overall SID cohort, other bacterial infections were reported in four patients at 6 months (four infections: dermatitis exfoliative (*n* = 1, 25.0%), flu-like illness (*n* = 1, 25.0%), sinusitis (*n* = 1, 25.0%), groin abscess (*n* = 1, 25.0%)), six patients at 12 months (six infections: appendicitis (*n* = 1, 16.7%), bronchitis (*n* = 2, 33.3%), sinusitis (*n* = 1, 16.7%), urinary tract inflammation (*n* = 1, 16.7%), paranasal sinus infection (*n* = 1, 16.7%)), one patient at 18 months (one infection: influenza (*n* = 1, 100.0%)), three patients at 30 months (four infections: bronchitis (*n* = 1, 25.0%), lung infection (*n* = 2, 50.0%), mycosis (*n* = 1, 25.0%)), and one patient at 36 months (one infection: COVID-19 (*n* = 1, 100.0%)). The overall bacterial infection rate was 9.1% (1/11 patients) at 36 months.

### 3.5. Training and Administration Health Resource Utilization

At inclusion, health resource utilization in the previous 12 months was reported. The number of nurse training sessions patients received regarding the correct method for administration of fSCIG was reported for 25 patients, with a mean of 1.8 ± 2.1 (range 0–4). Among the patients who self-administered (18/31 (58.1%)), the number of nurse training sessions for the correct administration of fSCIG was known for 14/18 (77.8%), with a mean of 2.1 ± 2.2 (range 0–5); half of these patients did not receive any training sessions. The mean number of visits nurses made to administer fSCIG to the patient at their home was 1.0 ± 1.5 (range 0–4). Similar data were observed across age subgroups. The median number of patient visits to the treating physician’s office was 4.0 (range 0–34). The per capita number of sickness days was 23.2 (range 0–365), and hospitalizations for the underlying SID condition occurred in 11/31 (35.5%) patients.

During follow-up, one patient required a nurse training session. Nurse visits (except for training) to the patient’s home to administer fSCIG were limited, with the highest frequency (1.3 ± 2.9 (range 0–8)) occurring at the 24-month follow-up interval. The median number of patient visits to the treating physician was 2.0 (range 0–4) at 18 months and 2.0 (range 0–24) at 24 months. The per capita number of sickness days was 5.5 (range 0–60) at 36 months and 31.2 (range 0–365) at 24 months. Most hospitalizations were related to the underlying SID condition, which accounted for 4% and 8% at the 6-month and 12-month follow-up, respectively, with no hospitalizations thereafter.

## 4. Discussion

The FIGARO study is the first prospective, multicenter, observational study evaluating fSCIG in patients with immunodeficiency (PID and SID) and various underlying conditions. This analysis of FIGARO provided insights into the real-world usage of fSCIG in patients with SID primarily due to hematologic malignancies, and the value of fSCIG treatment for improving the patient experience by allowing for individualized, patient-centric care. Treatments for SID, including IVIG and cSCIG therapy, can be associated with substantial treatment burden, including difficulties with managing hospital-based treatment or receiving frequent infusions [27,28]. In this analysis, fSCIG treatment was well tolerated in patients with SID and permitted flexibility in dosing and administration at home or in a medical facility (by the patient or a caregiver), during up to 36 months of follow-up.

During the 36-month follow-up, there was a trend toward increasing self-administration at home, with the majority of doses being given every 3–4 weeks. The median monthly dose of fSCIG, infusion volume (per patient), and maximum infusion rate remained constant over time. During follow-up, the IgG serum trough level in the SID cohort tended to increase. The majority of the patients required only one infusion site and were able to receive the full dose every 3–4 weeks at home. After the baseline visit, there were limited nurse training sessions and nurse visits at the patient’s home to administer fSCIG in the overall population and older adult patients (≥65 years of age). Additionally, patients were able to self-administer fSCIG at home with minimal technical problems.

fSCIG was well tolerated when administered at home or in a medical facility, with no ADRs reported during follow-up, despite patients in the SID cohort having a mean (SD) age of 61.4 (17.8) years and a high comorbidity burden. Premedication was used in only one patient across all follow-up visits. Although 32.3% of patients reported at least 1 ASBI event during the previous 12 months of study enrollment; no ASBI events were reported during follow-up. These findings support the effectiveness and tolerability of fSCIG across age groups regardless of the site of administration (home or medical facility).

Results of this prospective analysis were consistent with other observational and real-world studies that evaluated fSCIG use in patients with SID, where treatment with fSCIG was effective and well tolerated [25,29]. Patients were able to self-administer fSCIG at home every 3–4 weeks, using a single infusion site [25]. In a subset of patients from the SIGNS registry (Assessment of immunoglobulins in a long-term non-interventional study), the use of SCIG in patients with SID was found to be well tolerated, and infection rates were lower following treatment than in the 12 months prior to SCIG administration [25].

The current analysis illustrated the potential benefits of fSCIG as an alternative to other treatments for SID, including IVIG and cSCIG. fSCIG provides the option to individualize treatment, including choosing the site of care and number of infusion sites, which may reduce treatment burden and promote independence for patients with SID. By facilitating home-based treatment, fSCIG therapy can reduce the disruptions associated with scheduling IVIG treatment in a hospital setting, which can make it difficult to manage work, school, or social activities and may negatively impact patient HRQoL [27,30,31]. Most patients in the current analysis could receive their fSCIG infusion in a single infusion site every 3–4 weeks, in contrast to cSCIG therapy, which may require injections in multiple sites and a more frequent treatment schedule [6]. The frequency of administration of fSCIG in the current study was similar to the typical frequency of administration for IVIG, but without the need for venous access that can limit the use of IVIG in SID patients with compromised veins [6]. Previous studies have demonstrated reduced needle stick burden and improved patient well-being with fSCIG compared with cSCIG or prior therapies [28,30].

Limitations of this analysis include those inherent to any observational study. This was a prospective data collection study with no control arm. Univariate and multivariate analysis were not performed due to the small sample size, especially during the 36-month follow-up. Although data quality was checked on site, there is potential for missing data and inaccurate reporting of the frequency or severity of ADRs, because most infusions were administered at home by patients. FIGARO was conducted under the auspices of the ESID; therefore, the study was performed in a European cohort. The focus was on the countries where fSCIG was registered and reimbursed for the broad patient population (PID, SID, pediatric, and adult patients), and European countries were mainly eligible at that time. Experience with fSCIG at the European centers may not be generalizable to the experience outside Europe with different standards of care and clinical practice. Consistent with the diagnosis of hematological malignancy, most patients with SID were adults and older adults; therefore, it is difficult to distinguish if differences in the fSCIG utilization patterns were related to the age group or to the underlying indication for IGRT.

## 5. Conclusions

This study demonstrated that fSCIG positively influenced the treatment experience for patients with SID. Advantages of fSCIG include self-administration at home, better bioavailability than cSCIG [32], longer infusion intervals, and fewer ADRs in patients with SID. fSCIG was well tolerated when administered at home or in a medical facility, with no ADRs reported during follow-up, despite patients in the SID cohort having a mean age of 61.4 years and a high comorbidity burden. fSCIG provides the option to individualize treatment, including choosing the site of care and number of infusion sites, which may reduce treatment burden and promote independence for patients with SID.

## Figures and Tables

**Table 1:** Demographic, clinical, and treatment characteristics at inclusion.

Parameter	SID Overall Population ^a^ (*n* = 31)	18–64 Years (*n* = 15)	≥65 Years (*n* = 15)
Demographics			
Age (years), mean (SD)	61.4 (17.8)	52.5 (12)	74.1 (6.0)
Male, *n* (%)	19 (61.3)	8 (53.3)	10 (66.7)
Caucasian/White, *n* (%)	29 (93.5)	14 (93.3)	14 (93.3)
Clinical characteristics			
BMI (kg/m^2^), mean (SD)	25.4 (4.1)	25.5 (4.8)	25.6 (3.3)
Indication for IGRT, *n* (%)			
CLL	20 (64.5)	8 (53.3)	12 (80.0)
Indolent lymphoma	4 (12.9)	1 (6.7)	3 (20.0)
Other SID	7 (22.6)	6 (40)	-
B-NHL	1 (14.3)	1 (16.7)	-
Diffuse large B-cell lymphoma	1 (14.3)	1 (16.7)	-
Hodgkin’s disease	1 (14.3)	1 (16.7)	-
Hodgkin’s lymphoma after autologous transplantation	1 (14.3)	1 (16.7)	-
Lung transplantation, rituximab therapy	1 (14.3)	-	-
Rituximab	1 (14.3)	1 (16.7)	-
Immunosuppressive therapy for autoimmune disorders	1 (14.3)	1 (16.7)	-
Received chemotherapy, immunosuppressive therapy or supportive therapy, *n* (%)	29 (93.5)	13 (86.7)	15 (100.0)
Concomitant supportive therapy at initiation, *n* (%)			
Antibiotics	8 (25.8)	4 (26.7)	4 (26.7)
Corticosteroids	4 (12.9)	3 (20)	1 (6.7)
Expectorants	1 (3.2)	-	1 (6.7)
Inhalation therapy	4 (12.9)	2 (13.3)	2 (13.3)
PJP prophylaxis	14 (45.2)	4 (26.7)	10 (66.7)
Virostatics	13 (41.9)	5 (33.3)	8 (53.3)
Other supportive therapy	14 (45.2)	7 (46.7)	7 (46.7)
IGRT history ^b^			
IG route of administration ^c^, *n* (%)			
IV	17 (54.8)	9 (60.0)	7 (46.7)
SC	31 (100.0)	15 (100.0)	15 (100.0)
Total monthly dose of past and current IGRT (g), mean (SD)	23.1 (9.3)	24 (9.7)	23.2 (8.2)
Reason for prior treatment discontinuation and change to fSCIG therapy ^d^, *n* (% of total discontinued treatments)			
Tolerability	5 (21.7)	5 (38.5)	-
Patient request	6 (26.1)	2 (15.4)	3 (33.3)
Administrative	3 (13.0)	3 (23.1)	-
Other	9 (39.1)	3 (23.1)	6 (66.7)

^a^ Overall SID population includes 1 pediatric patient. This patient was not included in the SID sub-analysis. ^b^ Multiple responses possible. ^c^ Patients could have had both IV and SC. ^d^ Based on number of prior therapies, multiple responses possible. B-NHL, B-cell non-Hodgkin lymphoma; BMI, body mass index; CLL, chronic lymphatic leukemia; fSCIG, facilitated SCIG; IG, immunoglobulin; IGRT, immunoglobulin replacement therapy; IV, intravenous; PJP, *Pneumocystis jirovecii* pneumonia; SC, subcutaneous; SID, secondary immunodeficiency disease; SD, standard deviation.

**Table 2:** fSCIG dosing and infusion parameters at inclusion.

Parameter, Median (IQR)	SID Overall Population ^a^ (*n* = 31)	SID Overall Population ^a^ (*n* = 11)	18–64 Years (*n* = 15)	≥65 Years (*n* = 15)
	Inclusion	36 Months	Inclusion	Inclusion
fSCIG dose, g	30 (25–30)	30 (30–30)	30 (20–30)	30 (25–30)
fSCIG total monthly dose, g	30 (25–30)	30 (30–30)	30 (25–30)	30 (27.5–30)
fSCIG dose, g/kg/month	0.400 (0.333–0.500)	0.366 (0.348–0.462)	0.390 (0.304–0.484)	0.392 (0.350–0.501)
fSCIG infusion volume ^b^, mL	300 (250–300)	300 (300–300)	300 (250–300)	300 (250–300)
Right upper abdomen	300 (300–300)	300 (300–300)	300 (300–300)	300
Left upper abdomen	300 (100–300)	300 (300–300)	300 (300–300)	300
Right lower abdomen	300 (250–300)	300 (300–300)	300 (200–300)	300 (250–300)
Left lower abdomen	300 (200–300)	200	300	250 (200–300)
Right thigh	125	-	125	-
Left thigh	125	-	125	-
fSCIG maximum infusion rate, mL/h	280 (240–300)	300 (300–300)	240 (200–300)	300 (240–300)
Infusion location, *n* (%)				
Lower abdomen	12 (38.7)	4 (31.8)	3 (20.0)	9 (60.0)
Upper abdomen	9 (29.0)	6 (50.0)	7 (46.7)	1 (6.7)
Thigh	1 (3.2)	-	1 (6.7)	-
Unknown	9 (29.0)	2 (18.2)	4 (26.7)	5 (33.3)
Infusion interval, *n* (%)				
Every 2 weeks	1 (3.2)	1 (9.1)	1 (6.7)	-
Every 3 weeks	2 (6.5)	1 (9.1)	2 (13.3)	-
Every 4 weeks	22 (71.0)	9 (81.8)	9 (60.0)	12 (80.0)
Other	2 (6.5)	-	-	2 (13.3)
NA ^c^	4 (12.9)	-	3 (20.0)	1 (6.7)
Needle length, mm, *n* (%) ^d^				
9	8 (42.1)	-	5 (50.0)	3 (33.3)
12	10 (52.6)	2 (100.0)	5 (50.0)	5 (55.6)
20	1 (5.3)	-	-	1 (11.1)
Missing data	12 (38.7)	9 (81.8)	5 (33.3)	6 (40.0)
Needle diameter, 24 gauge, *n* (%) ^d^	19 (100.0)	9 (100.0)	9 (100.0)	10 (100.0)
IgG serum trough level, g/L	5.4 (4.6–7.3)	9.4 (7.5–9.5)	6.7 (4.6–7.4)	4.8 (4.3–5.6)

^a^ Overall SID population includes 1 pediatric patient. This patient was not included in the SID sub-analysis. ^b^ Sum infusion volume over all sites per patient. For *n* = 1, no IQR is available. ^c^ Not applicable as the patients received only one fSCIG infusion to date. ^d^ Values shown represent only those patients with available data. fSCIG, facilitated subcutaneous immunoglobulin; IgG, immunoglobulin G; IQR, interquartile range; NA, not applicable; SID, secondary immunodeficiency.

## Data Availability

The data sets generated and/or analyzed during the current study are available from the corresponding author on reasonable request, to researchers who provide a methodologically sound proposal. The data will be provided after their de-identification, in compliance with applicable privacy laws, data protection and requirements for consent and anonymization.

## Supplementary Materials

The following supporting information can be downloaded at https://www.mdpi.com/article/10.3390/cancers15184524/s1. Table S1: Rate of ASBI events and other bacterial infection events at inclusion.
