# Supplementary material for: Insights into Facilitated Subcutaneous Immunoglobulin Use in Patients with Secondary Immunodeficiency Diseases: A FIGARO Subgroup Analysis
Source: Cancers (Basel). 2023 Sep 12;15(18):4524. doi: 10.3390/cancers15184524 (PMC10526788; doi:10.3390/cancers15184524)
Supplement: Supplementary file 1 [file cancers-15-04524-s001.zip › cancers-2529031-supplementary.pdf]

Supplementary Table S1. Rate of ASBI events and other bacterial infection events at inclusion

Parameter	SID Overall Population[a] (n=31)	18-64 Years (n=15)	≥65 Years (n=15)
**ASBI events in the past 12 months, n (%)**			
No	21 (67.7)	11 (73.3)	10 (66.7)
Yes	10 (32.3)	4 (26.7)	5 (33.3)
**ASBI events (MedDRA code), n (%)[b]**			
Diarrhea, *Clostridium difficile*	1 (7.1)	-	-
Febrile neutropenia	2 (14.3)	1 (20.0)	1 (12.5)
Increased susceptibility to infections NOS	1 (7.1)	1 (20.0)	-
Infection of kidney, unspecified	1 (7.1)	1 (20.0)	-
Pneumonia	5 (35.7)	-	5 (62.5)
Pseudomonas infection NOS	1 (7.1)	-	1 (12.5)
Tonsillitis	2 (14.3)	1 (20.0)	1 (12.5)
Puncture site abscess	1 (7.1)	1 (20.0)	-
Total	14 (100.0)	5 (100.0)	8 (100.0)
**Other bacterial infection events, n (%)**			
No	20 (64.5)	9 (60.0)	10 (66.7)
Yes	11 (35.5)	6 (40.0)	5 (33.3)
**Other bacterial infection events (MedDRA code), n (%)[b]**			
Acute bronchitis	4 (14.8)	3 (15.8)	1 (12.5)
Gastroenteritis	1 (3.7)	1 (5.3)	-
Infection bacterial	2 (7.4)	2 (10.5)	-
Lower respiratory tract infection	3 (11.1)	-	3 (37.5)
Nasopharyngitis	1 (3.7)	1 (5.3)	-
Otitis	1 (3.7)	1 (5.3)	-
Parotitis	1 (3.7)	1 (5.3)	-
Pharyngitis	1 (3.7)	1 (5.3)	-
Pneumonia	3 (11.1)	2 (10.5)	1 (12.5)
Productive cough	1 (3.7)	1 (5.3)	-
Prostatitis	1 (3.7)	-	1 (12.5)
Pyelonephritis	1 (3.7)	1 (5.3)	-
Sinusitis	7 (25.9)	5 (26.3)	2 (25.0)
Total	27 (100.0)	19 (100.0)	8 (100.0)

[a]Overall SID population includes 1 pediatric patient. This patient was not included in the SID sub-analysis.
[b]Multiple responses possible.
ASBI, acute severe bacterial infection; MedDRA, Medical Dictionary for Regulatory Activities; NOS, not otherwise specified; SID, secondary immunodeficiency disease.
